# Defining Reablement in the Dutch Context: A Modified Delphi Study

**DOI:** 10.2147/JMDH.S522161

**Published:** 2025-05-23

**Authors:** Lise Elisabeth Buma, Ines Mouchaers, Sandra M G Zwakhalen, Stan Vluggen, Ton Satink, Silke F Metzelthin

**Affiliations:** 1 Department of Health Services Research, Care and Public Health Research Institute, Maastricht University, Maastricht, the Netherlands; 2 Living Laboratory in Ageing and Long-Term Care, Maastricht, the Netherlands; 3 Cicero Zorggroep, Brunssum, the Netherlands; 4 Academy of Nursing, Zuyd University of Applied Sciences, Heerlen, the Netherlands; 5 Research Group Neurorehabilitation – Self-Regulation and Participation, HAN_University of Applied Sciences, Nijmegen, the Netherlands

**Keywords:** restorative care, goal-oriented care, multidisciplinary collaboration, person-centered care

## Abstract

**Introduction:**

For the past decade, the Netherlands has been developing and implementing reablement programs to promote independence and to empower older adults’ autonomy. However, a key challenge remains the lack of clarity around the definition of reablement and its relations to usual care practices. Existing international definitions lack specificity to account for contextual differences, such as variations in healthcare systems and cultural norms. An operational definition is needed that not only fits the Dutch health and social care system and incorporates context-specific elements. This study extends the original conceptual definition by integrating these elements, offering clearer, more practical guidance for real-world application.

**Materials and methods:**

A modified Delphi study was performed to develop a definition of reablement that fits the Dutch health and social care system, extending beyond conceptual understanding. The study comprised three expert rounds and three Delphi survey rounds.

**Results:**

A total of 139 participants from Dutch health and social care, education, research, and representatives of clients and informal caregivers, participated. They evaluated statements in four sections: the target group, aims, type of care or support, and characteristics of reablement programs. Key discussions during the expert rounds focused mainly on 1) the target group, emphasizing the importance of involving individuals and their families, and 2) the characteristics of reablement, such as coordinating roles, team composition, and size. Input from the Delphi surveys and expert rounds led to the development of an operational definition for the Dutch context, agreed upon by 81% of stakeholders.

**Conclusion:**

The Delphi methodology proved valuable in identifying context-specific elements and incorporating expert perspectives, creating a culturally and contextually sensitive definition. This definition distinguishes itself from the international version by offering practical guidance on areas of application and interventions, with a focus on promoting social participation, well-being, and the involvement of the individual’s social network.

## Introduction

The increase in population ageing and high prevalence of chronic conditions are straining healthcare systems.^1^ Reablement offers a solution by promoting independence and empowering older adults’ autonomy through person-centered support and interventions.^2^ Currently, more than 15 countries have embraced the reablement approach, either as a national care policy or as a promising care concept. Still, its interpretation and application can vary greatly depending on the country and healthcare system in which it is implemented. To address this ambiguity, Metzelthin et al^3^ conducted a Delphi study in 2018 with 82 reablement experts from 11 countries to develop a conceptual, consensus-based, international definition of reablement:

Reablement is a person-centered, holistic approach that aims to enhance an individual’s physical and/or other functioning, to increase or maintain their independence in meaningful activities of daily living at their place of residence and to reduce their need for long-term services. Reablement consists of multiple visits and is delivered by a trained and coordinated interdisciplinary team. The approach includes an initial comprehensive assessment followed by regular reassessments and the development of goal-oriented support plans. Reablement supports an individual to achieve their goals, if applicable, through participation in daily activities, home modifications and assistive devices as well as involvement of their social network. Reablement is an inclusive approach irrespective of age, capacity, diagnosis or setting.

The international consensus based definition of reablement has been utilized as a foundation for policy and research in several countries in Western Europe and Asia.^4^ This definition outlines reablement in a broad, theoretical sense, and highlights its focus on enabling and empowering individuals. Due to contextual differences between and even within countries this conceptual definition lacks the specificity needed for practical application. For example, it requires more specific information about the target group, assessment tools, interventions, and processes. The ReAble Network^5^ advocates for developing country-specific operational definitions, based on the internationally accepted conceptual definition.^6^ Developing an operational definition for a specific context ensures that reablement practices are aligned with local needs and resources, such as the availability of trained professionals and cultural attitudes, thereby increasing their relevance and feasibility for practitioners and policymakers.

In the Netherlands, research on reablement has been conducted for over a decade. The focus has been on developing and implementing reablement programs, as well as evaluating their feasibility, stakeholder experiences and effectiveness in terms of client and care professional outcomes, and cost-effectiveness.^2^,^7^,^8^ With the introduction of a national policy program (Living, Support, and Care for the Elderly) by the Dutch Ministry of Health, Welfare, and Sport in 2022, reablement has gained increased national attention. It is presented as a sustainable solution to promote independence among older individuals and thereby reduce pressure on the healthcare system.^9^ This growing interest has stimulated numerous health and social care providers in the Netherlands to integrate reablement into their daily practices. However, there is ambiguity regarding the definition of reablement and how it relates to usual care practices, with many organizations left to navigate its implementation without clear guidance. An operational definition is needed that not only fits the Dutch health and social care system but also refines and build upon existing definitions of reablement. This study progresses the original conceptual definition by incorporating context-specific elements and drawing input from relevant stakeholders. By doing so, it aims to create a more practical and context-sensitive definition, offering clearer guidance for real-world application. The term “operational definition” in this study refers to guidance for policy and practice that extends beyond a conceptual definition, outlining the operationalization of reablement in practice in terms of its target group, aims, type of care or support, and characteristics. We aimed to develop an operational reablement definition by incorporating the perspectives of various stakeholders across practice, policy, and research.

## Methods

### Study Design

We conducted a modified Delphi study. It deviated from the traditional Delphi method by incorporating additional elements alongside the expert panel, such as the scientific literature and stakeholder input, obtained through open-ended questions.^10^ The Delphi technique is used to identify the collective opinion of experts and to detect agreement.^11^ In our Delphi study, three rounds of expert panel meetings and three Delphi survey rounds were conducted between April 2024 and September 2024 following the recommendations for Conducting and Reporting of Delphi Studies (CREDES) to increase robustness.^12^

### Participants and Recruitment

#### Expert Panel

Purposive sampling^13^ was used to invite stakeholders to form an expert panel, to provide informed opinions and insights on reablement based on their experiences. The experts were selected based on their expertise in reablement, covering health and social care professionals. Recruitment was conducted through the Dutch reablement network, ensuring the inclusion of professionals from organizations considered frontrunners in reablement implementation. This included four project leaders, a policy advisor for the municipality, members of reablement teams (ie, occupational therapist, physiotherapist, community nurse, and a district linking pin), as well as representatives of clients and informal caregivers. This approach aimed to ensure a panel with relevant knowledge and practical experience in diverse settings with diverse backgrounds.

#### Survey Participants

Online surveys were used in all three Delphi rounds. For these online survey rounds, a large group of stakeholders across the Netherlands was recruited through word of mouth, email, and social media. This large group included a mix of stakeholders and the members of the expert panel, comprising all individuals involved in reablement or those who have experienced it firsthand – such as clients, informal caregivers, social workers, nursing staff, therapists, management, educators, researchers, and others – without any specific eligibility criteria.

Before the start of the study, all participants were provided an information letter, that stated the study’s background, objectives, and participation information. Informed consent was obtained prior to the start of the study, including publication of anonymized responses/direct quotes.

### Data Collection

**Figure 1:**
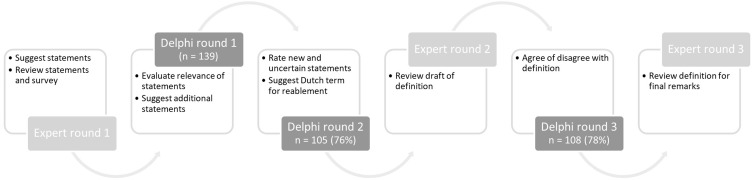
The modified Delphi process consisting of three online Delphi rounds and three expert panel meetings.

The surveys were conducted using the online survey program Qualtrics^14^ Background information such as gender, age, education, job title, primary field of work, work area, working experience, experience with reablement, and knowledge of reablement was collected from all participants. The research team, consisting of the authors LB, IM, SZ, SV, TS, and SM, was responsible for all aspects of the Delphi process, including preparation, execution, analysis, and reporting of the study. Figure 1 shows an overview of the steps of the Delphi process including an overview of the total number of respondents per round.

#### Expert Round 1 (April - May 2024)

A literature search was conducted to gather existing descriptions of reablement from scientific papers. Drawing on the international conceptual definition by Metzelthin et al^3^ the research team identified statements related to the target group, aims, characteristics, and components of reablement. These were used to create a preliminary set of statements. Then, an online survey was conducted with the expert panel, who were asked to generate statements about the target group, aims, characteristics, and components of reablement through open-ended questions, without prior knowledge of the literature-based statements. The research team incorporated the experts’ contributions and the findings from the scientific papers and the international Delphi study to refine the statements. The resulting first survey was divided into four sections – target group, aims, type of care or support, and characteristics. The expert panel subsequently reviewed these statements, offering feedback and suggestions. The research team discussed all the feedback, which included recommendations on survey structure, language use, and the inclusion of examples. The final version of the survey included 80 statements.

#### Delphi Round 1 (May – June 2024)

**Figure 2:**
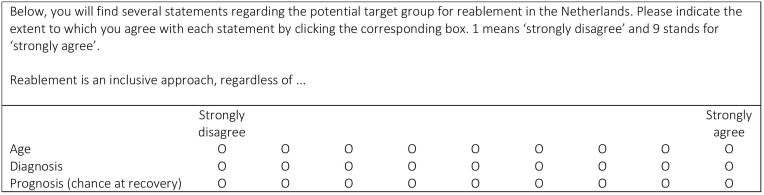
Sample survey questions for the target group prompted the participants to provide their responses based on their ideal scenario for reablement in practice.

In the first survey, the participants were asked to evaluate the 80 statements using a nine-point Likert scale, where higher scores corresponded to greater agreement on that the statement was relevant to the question (see Figure 2). The participants were encouraged to suggest additional statements if they felt important topics were missing and were able to respond to the statements through open-ended questions. These additional comments were thoroughly reviewed by two members of the research team (LB and SM). They were subsequently used in round 2 to rephrase and refine specific statements and text and were also incorporated into discussions with the expert panel during round 2. Statements with any ambiguity were reassessed in the subsequent round.

#### Delphi Round 2 (June – July 2024)

All participants from round 1 were invited for round 2. Two reminders were sent to increase the response rate. The adapted survey included additional statements as suggested by the participants in round 1. In addition, the statements with ambiguity from the first survey were evaluated using a binary response option (include versus do not include in the definition).

After collecting all the responses, the research team created a draft of the definition based on the statements. The text was divided into sections on the aims, target group, the individual’s needs, and characteristics of reablement.

#### Expert Round 2 (July 2024)

The draft was presented to the expert panel, who provided feedback during a two-hour online meeting led by two members of the research team (LB and SM). During the meeting, the panel was divided into two groups. Each group reviewed the text sections of the definition to refine nuances and to ensure the precision of the wording and terminology used. Notes were taken by the researchers. After both groups had discussed the definition, they reconvened and shared their findings with the other group. During each step, the panel members were allowed to provide clarifications, add nuances, and make further contributions. The outcomes were collectively reviewed afterwards.

The draft was subsequently revised and refined by the research team based on the suggestions of the expert panel. Within the research team, agreement was also sought regarding the use of certain terms or concepts and keeping all of the participants’ suggestions in mind.

#### Delphi Round 3 (July – September 2024)

In the third round, the refined definition was shared with the participants form Delphi rounds 1 and 2. They were invited to indicate their agreement with the proposed definition or to suggest any modifications if they did not agree. Two reminders were sent to increase the response rate. After collecting all the responses, the research team revised the draft of the definition based on the feedback received. The, the revised draft was shared with the expert panel via email, allowing them to offer any final comments. These were incorporated into the document, resulting in the final version.

### Data Analysis

All analyses were performed using SPSS Statistics version 28.0.1.1.^15^ Descriptive statistics were used to analyze the background characteristics of the participants (ie, frequencies, percentages, means, and standard deviations [SDs]). For the questions employing the Likert-scale in Delphi rounds 1 and 2, median scores and inter-quartile ranges (IQR) were used to assess relevance and the level of consensus, respectively. Statements were deemed relevant if they achieved a median score of 7–9 and an IQR of ≤2.^16^ Statements with a median score of 1–3 and an IQR of ≥2 were considered to be less relevant and lacking consensus and were subsequently excluded.^16^ The remaining statements were considered uncertain. For questions employing a binary response option (include versus do not include in the definition, agree versus disagree with the definition draft), a threshold of 75% was established to determine agreement.^12^ This threshold was chosen based on established Delphi methodology in healthcare research, balancing inclusivity with methodological rigor.^17^ Responses were not weighted by experience or primary field of work; all panel members’ inputs were equally considered to ensure broad representativeness.

The notes taken during various steps of the process were reviewed and discussed by the research team, and then thematically summarized to identify key messages and insights. In addition, to capture changes in expert opinion and identify evolving themes, qualitative analysis was applied to responses, key insights were reviewed by the research team. This allowed us to capture and interpret significant topics and themes, providing a deeper understanding of the data evolving consensus on the definition, particularly given the challenges of direct statistical comparisons across rounds in Delphi studies.^18^

### Ethical Considerations

The study was reviewed and approved by the Faculty of Health, Medicine & Life Sciences (FHML) Research Ethics Committee of Maastricht University in the Netherlands, under approval number FHML-REC/2024/014. The study was not subject to the Dutch Medical Research Involving Human Subjects Act (WMO). All participants received information about the study’s purposes, provided informed consent, and had the right to withdraw from the study at any moment. All data were pseudo-anonymized and stored on the research server of Maastricht University and only accessible to the members of the research team.

## Results

In total, 139 participants across the Netherlands participated in round 1 of the Delphi study; of these, 105 (75.5%) participated in round 2, and 108 (77.6%) participated in round 3. An overview of the participants’ background information is presented in Table 1. Of the participants, 81.3% were primarily employed in healthcare, 7.2% within social care, 4.3% in education or research, and 7.2% in other sectors (eg, health insurer, client, and caregiver representatives). Additionally, 37.4% held management-related positions (eg, manager, policy advisor, project leader), while 54% were practitioners within health and social care (eg, registered nurses, occupational therapists, and social workers). The participants had an average of 8.3 years (SD 7.8) of work experience in their current occupation, an average of 2.8 years (SD 4.2) of experience with reablement and self-rated their knowledge of reablement with an average 7.1 out of 10 (SD 1.5).

**Table 1:** Background Information of the Participants (n = 139)

	Delphi participants (n = 139)
**Age (years), mean (SD)**	44.8 (13.2)
**Gender, n (per cent)**	
Men	19 (13.7)
Women	119 (85.6)
Non-binary	1 (0.7)
**Educational level^a^, n (per cent)**	
Low	16 (11.5)
Intermediate	72 (51.8)
High	51 (36.7)
**Occupation, n (per cent)**	
** Healthcare professionals**	70 (50.4)
Nursing professionals^b^	31 (22.3)
Medical and allied health professionals^c^	35 (25.2)
Advisors and case management^d^	4 (2.9)
** Social care professionals**^e^	5 (3.6)
** Management**	52 (37.4)
Manager	21 (15.1)
Policy and strategy officer or advisor	17 (12.2)
Project leader	14 (10.1)
** Other**	12 (8.6)
Researcher/educator	7 (5.0)
Client or formal/informal caregiver representative	4 (2.9)
Management assistant	1 (0.7)
**Working experience in current occupation (years), mean (SD)**	8.3 (7.8)
**Experience with reablement (years), mean (SD)**	2.8 (4.2)
**Self-rated knowledge of reablement (1–10)**^f^, mean (SD)	7.1 (1.5)
**Primary field of work, n (per cent)**	
** Healthcare sector**	113 (81.3)
Rehabilitation care	12 (8.6)
Institutionalised long-term care	36 (25.9)
Hospital care	1 (0.7)
Community care	49 (35.3)
** Social care sector**	10 (7.2)
** Education/research**	6 (4.3)
** Other**^g^	10 (7.2)

**Notes**: ^a^ Low: primary education, lower secondary education; Intermediate: intermediate vocational or higher secondary education; High: higher vocational education, university. ^b^ Registered nurse, certified nurse assistants, nurse assistants. ^c^ Occupational therapist, physical therapist, elder care physician/nurse practitioner, speech therapist, psychologist. ^d^ Dementia case managers, behavioural advisor, informal care consultant. ^e^ Social worker, district linking pin, day care services worker, social prevention worker. ^f^ Higher score indicates higher self-rated knowledge. ^g^ Retired, ICT, member informal care and client council, welfare, health insurer, board member, self-employed.

**Abbreviation**: SD: standard deviation.

**Figure 3:**
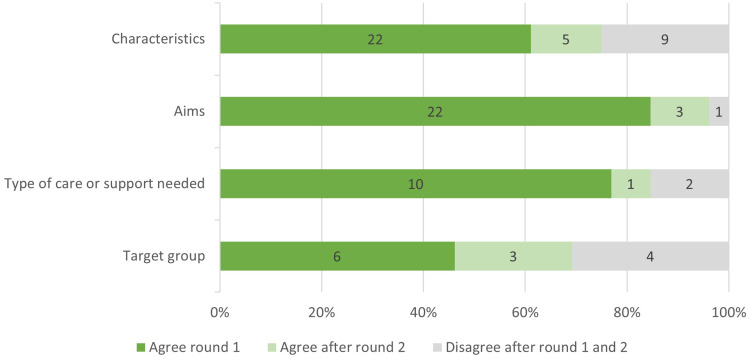
Proportion of statements related to each section of the definition that did or did not reach agreement after rounds 1 and 2 of the Delphi study. The numbers indicate the number of statements.

In the following sections, we present the findings and participant discussions for each part of the definition on reablement (ie, the target group, aims, type of care or support needed, and characteristics) and present the final phrasing of each section. Figure 3 shows the proportion of statements related to each part of the definition that did or did not reach agreement in the Delphi rounds. A scoring summary of all statements of Delphi rounds 1 and 2 is provided in Appendix 1.

### Target Group

#### Delphi Rounds

In round 1, six target group–related statements were identified as relevant. Five were rated as uncertain (median score 6–7, IQR ≥2: prognosis, cognitive functioning, psychological functioning, learning ability, and motivation) and re-evaluated in round 2 (see Appendix 1). Agreement was reached for “cognitive functioning” (75.2%), while the other four were excluded. The participants also suggested two new statements (“cultural background/ethnicity” and “social support availability”), both of which were included after round 2 scoring. Feedback from open-ended questions during round 1 highlighted nuances, such as using the term “diagnosis” and addressing both the individual and their family:

The individual and their family are the center of the treatment, and everything revolves around them. – Participant survey, round 1

#### Expert Panel Rounds

During each expert panel meeting in round 2, the target group for reablement was a key focus. Stakeholder suggestions were discussed alongside considerations about the intended population and the optimal wording to describe them. While the panel explored the characteristics of an ideal target group, the experts found it challenging to define this group precisely, resulting in a lack of clear criteria. They noted that eligibility is often determined by a combination of factors:

Reablement is fundamentally always an option. It often involves a combination of factors that may render reablement no longer feasible; a single factor does not provide sufficient information on the matter. – Client and informal caregiver representative

In round 2, the expert panel discussed the term “care avoiders”, deeming it inappropriate as it relates more to willingness to engage with reablement. They emphasised that this group requires a different approach and excluded it from the definition. The panel also stressed the importance of considering individuals and their social network as a unit in the definition. They noted that learning ability should not determine eligibility for reablement if the social network can provide support. Additionally, they emphasised that reablement requires the target group to identify a specific goal or support need, recommending this be included under characteristics. Motivation, viewed as a defining factor for the success of reablement, was included in the definition under the type of care or support needed.

#### Final Definition of the Target Group

The final text regarding the target group, as agreed upon following the surveys and expert panel meetings, is shown in Box 1.

**Box 1:** Final Definition Text for the Target Group for Reablement in the Netherlands

Reablement is an approach suitable for anyone with a care, support, and/or welfare need, regardless of age, culture, diagnosis, or level of physical, cognitive, and daily functioning. This approach can be applied both at home and within care organizations (such as hospitals, nursing homes, or rehabilitation centers) and is accessible to individuals living alone or with others. Reablement always focuses on both the individual and their surrounding social network.

### Aims

#### Delphi Rounds

In round 1, 22 statements related to the aims were identified as relevant to the definition. Three were rated as uncertain (median score 5–8, IQR ≥3: “reducing health and social care costs”, “facilitating discussions about the future”, and “enhancing informal caregivers’ confidence”) and re-evaluated in round 2 (see Appendix 1). Agreement was reached for including “facilitating discussions about the future” (86.7%) and “enhancing informal caregivers’ confidence” (79.0%), while the remaining uncertain statement was excluded. The participants in round 1 suggested a new statement – “deploying professional assistance where it is most needed” – but it was not included after round 2 scoring. Feedback from open-ended questions in round 1 offered further suggestions, such as removing certain statements or clarifying nuances. For example, some participants found the statement “reducing health and social care costs” to be framed too negatively and noted that some questions were difficult to assess.

I find it somewhat challenging to fill in. For example, I don’t think the goal is to relieve either professional care or family members, but that this can be a beneficial side effect. – Participant, round 1

#### Expert Panel Rounds

During the expert panel meetings, stakeholder suggestions were reviewed alongside considerations regarding the aims, focusing primarily on the precise wording of statements and the final text. For example, “participation” was revised to “social and community participation”. Some phrases were found to be too negative or insufficiently precise and were adjusted in consultation with the panel across all three rounds. The experts highlighted that the initial draft in round 2 did not sufficiently differentiate reablement from usual care in its aims. This was addressed in the final version, emphasizing the importance of focusing on meaningful activities, relationships, and participation, among other changes. The expert panel also stressed the importance of the social network and social care, noting that early drafts in round 2 were predominantly oriented towards health care, leaving these aspects underrepresented. Furthermore, they indicated the significance of prevention as a key component of reablement, recommending its explicit inclusion in the definition. These adjustments were incorporated into the aims and integrated across other sections of the text.

#### Final Definition of the Aims of Reablement

The final text regarding the aims, as agreed upon following the surveys and expert panel meetings, is shown in Box 2.

**Box 2:** Final Definition Text for the Aims of Reablement in the Netherlands

Reablement aims to enhance individuals’ autonomy and self-reliance, to improve their quality of life, and to enable them to remain in their living environment for as long as possible. It increases independence in both daily activities and activities and relationships that are meaningful to them. Moreover, reablement promotes social and community participation. Finally, reablement facilitates appropriate care and support, thereby potentially reducing the demand for professional care and support.

### Type of Care or Support Needed

#### Delphi Rounds

In round 1, ten statements regarding the type of care or support needed were deemed to be relevant to the definition. Two statements – related to “care avoiders” and “acute needs” – were rated as uncertain (median score 6–7, IQR =3) and re-evaluated in round 2 (see Appendix 1). Both were ultimately scored not relevant for inclusion by 60.0% and 67.6% of participants, respectively. Additionally, a new statement suggested in round 1 – concerning short-term residence in institutions such as hospitals, care homes, or rehabilitation centers – was included following round 2 scoring. During round 1, a participant provided feedback on the type of care or support needed, indicating that they did not give maximum scores on the Likert scale because it strongly depends on the implementation of reablement.

It [regarding the type of need for which reablement is implemented] strongly depends on how reablement is implemented. I believe that some needs do not necessarily require an interdisciplinary team with various healthcare professionals, as that might be excessive. – Participant, round 1

#### Expert Panel Rounds

During the expert panel meetings, stakeholder suggestions were reviewed alongside considerations about the type of care and support needed. Discussions also focused on refining the wording of specific statements and the final text, including examples related to “support needs”, “well-being needs”, and “preventive needs”. A key topic in round 2 was identifying the types of needs suitable for reablement, although no clear consensus emerged. For example, there was debate about whether reablement is only appropriate for complex needs and how such needs should be classified as complex. The panel also discussed the roles of individuals and their social networks, concluding that while individuals should lead the process, their networks – often overburdened – must be considered as well. This reinforced the view that individuals and informal caregivers should be regarded as a unit. The panel emphasised that reablement must integrate the perspectives of both individuals and their informal caregivers. To reflect this, adjustments were made across multiple sections of the text in rounds 2 and 3 to ensure this emphasis was clearly conveyed in the final definition.

#### Final Definition of Type of Care

The final text regarding the type of care or support needed, as agreed upon following the surveys and expert panel meetings, is shown in Box 3.

**Box 3:** Final Definition Text for the Type of Care or Support Needs Eligible for Reablement in the Netherlands

Reablement can be broadly applied to address both simple and complex issues related to independence and participation. It can assist with existing concerns or help prevent (future) issues. Reablement may focus on the following types of needs: Care needs (such as community nursing and paramedical care); Support needs (such as assistance with household tasks and transportation services); Well-being needs (such as day activities and guidance); or Combination of the above needs. Reablement is also employed to balance the burden and resilience of the individual and their social network. While receiving informal care is not a prerequisite, it can either support or hinder the achievement of the established goals. Furthermore, motivation plays a crucial role in the success of reablement; for example, an individual may experience significant limitations but can still achieve success through strong motivation, or conversely, may struggle despite experiencing fewer limitations.

### Characteristics

#### Delphi Rounds

In round 1, 22 statements about the characteristics of reablement were deemed relevant to the definition. Nine statements were rated as uncertain (median score 4–8, IQR ≥3), including those addressing the programme’s end, the core team, and the role of the individual and their social network, and were re-evaluated in round 2 (see Appendix 1). Four statements – “it stops when the individual’s goals have been achieved”, “a coordinator is designated within the core team”, “the individual is part of the core team”, and “the individual appoints someone from their social network to be part of the core team” – were deemed relevant for inclusion, while the other five were excluded. In round 1, the participants also suggested four new statements related to characteristics: “it can be monodisciplinary”, “the core team specifies which expertise is needed to achieve the goals”, “the individual takes on a coordinating role (possibly with professional support)”, and “the informal caregiver takes on a coordinating role (possibly with professional support)”, as well as “the individual is monitored even after goal attainment”. Among these, only “the core team specifies which expertise is needed to achieve the goals” was included following round 2 scoring. Feedback from the open-ended questions during round 1 raised questions about who should hold the coordinating role within the core team. Additionally, one participant highlighted the importance of regular evaluations with the individual to ensure sustained results.

A key component of the program is that after achieving the goals, there should be monthly check-ins to assess progress, with evaluation, adjustment, and follow-up being essential. After all, circumstances in the individual’s life and their environment are always changing. Restarting the process does not seem like a viable option. Someone from social care must remain closely involved, with a clear mandate for observation. – Participant, round 1

#### Expert Panel Rounds

During the expert panel meetings, stakeholder suggestions from each round were reviewed, with particular attention to the wording of specific statements and the final text (eg, “behavior change” can have a negative connotation). In round 2, discussions focused on the operationalization of reablement and the characteristics essential for its implementation. The experts highlighted the importance of a comprehensive intake process that accounts for well-being and participation. They also stressed the significance of involving the individual as the owner of the process and aligning interventions with the individual’s capabilities. The panel also discussed the value of providing tailored care and support, noting that achieving reablement goals does not always require a large, multidisciplinary team. These considerations were incorporated into the final text, with adjustments made in rounds 2 and 3 to ensure these principles were clearly reflected.

#### Final Definition of the Characteristics of the Approach

The final text regarding the characteristics, as agreed upon following the surveys and expert panel meetings, is shown in Box 4.

**Box 4:** Final Definition Text for the Characteristics of Reablement in the Netherlands

Reablement is implemented by an interdisciplinary team comprising professionals from both health and social care (core team). The individual receiving care and/or support is considered to be a member of the core team and may appoint someone from their social network to participate as well. Collaboration among various disciplines and sectors facilitates a broad, integrated approach. Team members are trained in the principles of reablement and possess the necessary competencies to deliver a reablement program. One team member assumes a coordinating role. The reablement process begins with a comprehensive needs assessment that takes a holistic view of the individual’s needs, wishes, and capabilities, alongside those of their social network. Following this, the individual (and/or their informal caregiver) formulates goals tailored to their specific situation. The core team collaborates with the individual to develop a plan aimed at achieving these goals, ensuring that the individual and their informal caregiver(s) maintain reliance throughout. This plan outlines goals, interventions*, and responsibilities. Regular evaluations and adjustments of the plan ensure the best possible outcomes are achieved. The level of care and support gradually decreases throughout the reablement process until the goals are met. Reablement is temporary in nature; if necessary, referrals to (long-term) care and support can be made upon completion. *The following interventions and techniques may be employed to achieve the established goals: Awareness and empowerment – for example, education, positive reinforcement and feedback, motivational interviewing, goal setting and planning, self-monitoring, and reflection; (Re)Learning cognitive, physical, emotional, and social skills – for example, memory training, fall prevention, exercise programs, coping with stress and emotions, and establishing and maintaining social connections; and Applying internal and external compensation strategies – for example, advising on and teaching the use of assistive devices, adapting tasks or environments, implementing strategies to enhance the balance between burden and resilience, and self-management.

### Refining the Definition

After drafting the initial complete definition text, 88 (81.5%) of the 108 participants in round 3 agreed with the first draft (see Appendix 2). The 20 participants (18.5%) who did not agree with the definition were given the chance to refine the text. Their comments addressed aspects such as language use, the rationale, and the focus of reablement. Table 2 provides details on the suggestions made and how they were incorporated into the final version of the text.

**Table 2:** Comments Given by Participants During Delphi Round 3 That Led to Adjustments in the Definition

Summary of Comments	Adaptations
Several experts mentioned that the phrase “actively participating in care” suggests that a person continues to receive care after reablement, whereas the core aim is to enhance self-reliance, empowerment, and personal control.	We decided to use the term “culture change” and added that “reablement encourages to help individuals learn to help themselves (again)”.
One expert found the phrasing regarding the application of reablement in a “familiar living environment” to be too restrictive, as the approach is also suitable for use in nursing homes or temporary living arrangements.	We changed the term to “living environment” to also include other settings.
An expert suggested adjusting the wording to make it clear that the individual sets their own goals.	We specified the fact that the goals are set “together with the individual”.
Several experts indicated that it is not sufficiently clear when reablement should be initiated.	We added that reablement can be initiated for everyone “with a care, support, and/or welfare need” and provided examples in the text.
One expert specifically mentioned that the temporary nature of reablement as an intervention is not adequately emphasised, creating the impression that it is a long-term process that may lose sight of its primary goal.	We added the phrase “reablement is of a temporary nature”.
An expert suggested that prevention could also be included as a principle.	We added this to the aims of reablement.
One expert noted that the text did not sufficiently reflect the equality between the individual or their representative and the reablement team.	We adjusted the sentence structure to better reflect this equality in the section on the characteristics of reablement.
Several comments were made regarding informal care, including the point that reablement is not intended for the informal caregiver, but rather should focus on the individual.	We refined this by clarifying that within reablement, “attention is also given to (potentially) overburdened informal caregivers, to help the individual remain in their living environment”.

Some experts’ feedback focused more on the reablement mindset rather than the specific content of reablement as a program. Additional suggestions led to a restructuring of the text to improve the readability. After the research team revised the text, it was forwarded to the expert panel for a final review. Three experts provided additional remarks, primarily focused on readability and word choice (eg, “needs assessment” instead of “intake”, with one expert suggesting the addition of more examples to improve operationalization). In response, the research team further refined the text to ensure a more concise and polished formulation, resulting in the final definition presented in Appendix 3.

## Discussion

The expert and Delphi rounds led to the development of the operational definition of reablement in the Netherlands presented in Appendix 3, which was agreed upon by 81.5% of the stakeholders. The key discussions focused on the target group, such as the importance of involving individuals and their families, and the characteristics of reablement, including the coordinating role, team composition, and size.

The operational definition closely aligns with the international definition by Metzelthin et al^3^ when considering the scope and focus of both definitions. While international definitions of reablement often emphasize short-term, goal-oriented support aimed at improving independence in daily functioning,^19–21^ the Dutch definition distinguishes itself by offering more detailed and practical guidance on the implementation of elements such as areas of application and interventions that can be used to reach the clients’ goals. Notably, in line with the international definition^3^ no specific target group was identified, highlighting the broad applicability of the approach. Moreover, the Dutch definition particularly focuses on social participation and connection, expanding its focus beyond independence to the individual’s social network and well-being. This reflects a move from individual autonomy to “meaningful functioning”, offering a more holistic and person-centred interpretation of goals. This aligns with trends in Dutch healthcare that focus on integrated care, prevention, and person-centered approaches to support older adults in living fulfilling, independent lives while participating in society.^9^,^22^ The focus on social connectedness and participation further positions the Dutch reablement definition as especially relevant within today’s policy context, reflecting a shift towards more holistic care that integrates formal services with community and social support to enhance overall well-being and resilience. These trends highlight that care-related challenges do not always need to be solved solely through formal care; they can also be addressed through social care and the support of the individual’s social network. The evolution of the concept and operationalization of reablement towards the social domain and well-being is evident globally, as seen in the development of reablement practices over time. Initially, reablement was more focused on functional recovery from a biomedical perspective – for example, “reabling” individuals back to work – and often aimed at specific conditions such as arthritis or cerebral palsy.^23^ However, over the years, the importance of social care and overall well-being has emerged more often in the reablement literature. This is not surprising, given the shifts in broader perspectives on health throughout the world, such as the World Health Organization’s concept of “Healthy Ageing”.^24^ Healthy Ageing is defined as the process of promoting and preserving functional ability to support well-being in later life. It focuses on enhancing an individual’s capacity to engage in activities that are meaningful to them, enabling them to “be” and “do” what they value.^24^

Our data also revealed a distinction between reablement as a mindset and reablement as an intervention, with the participants discussing that fostering this reablement mindset is a prerequisite for successful implementation of the intervention. The distinction between mindset and intervention has also emerged within the reablement literature, with Metzelthin et al^25^ also making a distinction between grounded service models and time-limited intervention programs in reablement principles. They argued that reablement service models aim to build capacity and foster environments enabling professionals to assist individuals in engaging in meaningful activities, thereby cultivating a reablement mindset rather than delivering time-limited interdisciplinary reablement interventions. This distinction influences the intended recipients and providers of reablement – for example, in how providers are trained, or services are structured, it reflects a move from “doing reablement” as a programmatic intervention to “being reablement” as a holistic, integrated approach within care systems. Moreover, Vluggen et al^26^ highlighted the importance of first cultivating the right mindset before implementing the program with clients. Attempting to implement the program without focusing on the right mindset risks reducing it to a mere checklist, where the core principles of reablement are not fully realized. Conversely, focusing only on mindset without offering adequate descriptions and guides of the approach for its implementation and sustainability may fail to achieve lasting change.

Our study presents several important insights with implications for both practice and research. First, while the operational definition provides a guide outlining the operationalization of reablement in practice, it does not provide information on how it relates to existing forms of care, which is necessary to clarify reablement’s value and to receive the necessary resources and support to implement reablement into standard care. To achieve this, it is important to compare reablement with existing interventions such as traditional home care or outpatient rehabilitation. One example of how this comparison can be made is through patient journeys, which could illustrate key differences in outcomes and approaches.^27^ This comparison can highlight both the differences and similarities, helping to clarify reablement’s value and its potential to become part of standard care. Second, while the conceptual international definition helps to anchor what reablement is in a broad, theoretical sense, the operational definition describes reablement in practice by outlining specific actions, interventions, and processes. Together, these definitions offer both a theoretical understanding of reablement and practical guidance for its application. For policymakers, these definitions ensure consistent and clear guidance that can support the creation of policy aligned with the principles of reablement. For practitioners, they provide a tangible structure for defining roles, responsibilities, and measurable outcomes, helping to translate theory into effective practice. For example, the definition can inform the development of outcome sets aligned with core reablement goals—such as meaningful functioning, social participation, and self-efficacy in daily activities—measured through validated instruments and narrative tools. It may also guide the selection of process indicators, including goal-setting quality, interdisciplinary collaboration, and client involvement, to assess feasibility and implementation. Third, the results of our study suggest that future reablement research should focus on outcomes related to well-being and social connectedness, as these areas are underexplored to capture the benefits of reablement and are often emerging themes in qualitative research.^28^ This could provide a valuable addition to the existing outcome measures used within reablement, as there are often doubts as to whether these measures fully capture the impact of reablement.^29^ Fourth, a critical consideration for future studies is to evaluate the real-world applicability of the definition across different healthcare settings. Qualitative research could assess how well the Dutch definition translates to different contexts and its level of adoption in practice. Our study provides a valuable example for other countries facing difficulties in aligning reablement practices due to conceptual ambiguity arising from varying interpretations. Our study can offer a blueprint for countries looking to develop their own context-sensitive definition, allowing them to view it through their own lens and take cultural differences and variations in healthcare systems into account. By systematically engaging diverse stakeholders – for example, through the Delphi method – it is possible to generate context-sensitive insights into reablement’s goals, target group, core characteristics, and interventions. This process can help to bridge gaps in understanding and align practices with the broader philosophy of reablement. In doing so, it strengthens the foundation for effective implementation, collaboration, and robust evaluation of reablement services.

A major strength of our study is its robustness: we followed the CREDES guidelines for conducting and reporting our research.^12^ Additionally, the Delphi methodology allowed for an iterative process to continuously refine and clarify the definition, incorporating data triangulation through expert and survey input to ensure a well-considered outcome. By engaging with a large group of stakeholders, we enhanced the applicability and relevance of our findings. The Delphi approach also ensured the definition was sensitive to the specific cultural and contextual aspects of the Dutch healthcare system. However, our study is also subject to certain limitations. For example, selection bias may have occurred, as most participants were from the healthcare sector and had a higher education, with a relatively small number from social care and those with lower levels of education. Consequently, the recommendations and conclusions drawn from the study might be less applicable or generalizable to these underrepresented populations and may not produce the same results in a in a future study with a comparable sample. Finally, reablement is a relatively new concept in the Netherlands, as confirmed by the average of 2.8 years of experience reported by participants. This may have affected the depth of understanding and the overall generalizability of our findings, as reablement does not have an established standard in the Dutch healthcare system.

## Conclusions

In conclusion, our study developed an operational definition of reablement in the Dutch healthcare context using the modified Delphi methodology. This approach proved to be a valuable tool for identifying context-specific elements and incorporating the experiences of experts, facilitating the creation of operational definitions that are both culturally and contextually sensitive. The developed definition serves as a guide for operationalizing reablement in practice, extending beyond a conceptual definition to offer concrete, actionable guidance. By engaging with a diverse group of experts, we sought to capture context-specific insights that could clarify key characteristics and offer practical guidance for its implementation. Future research should examine the adoption of the definition, assess its applicability across diverse healthcare settings, and identify barriers to its widespread implementation. Despite some limitations, the definition may contribute to both the theoretical and practical understanding of reablement. It could serve as a foundation for further research and policy development, helping to standardize and support the integration of reablement in the Netherlands and provide an example for other countries aiming to develop context-sensitive definitions.

## Data Availability

The data supporting the findings of this study are available upon request from the corresponding author.

## Abbreviations

CREDES, Conducting and Reporting of Delphi Studies;^12^ SD, Standard deviation; IQR, Inter-quartile range.
